# ROBL-II at ESRF: a synchrotron toolbox for actinide research

**DOI:** 10.1107/S1600577520014265

**Published:** 2021-01-01

**Authors:** Andreas C. Scheinost, Juergen Claussner, Joerg Exner, Manuel Feig, Stefan Findeisen, Christoph Hennig, Kristina O. Kvashnina, Damien Naudet, Damien Prieur, Andre Rossberg, Moritz Schmidt, Canrong Qiu, Patrick Colomp, Cedric Cohen, Eric Dettona, Vadim Dyadkin, Thorsten Stumpf

**Affiliations:** a The Rossendorf Beamline (BM20), European Synchrotron Radiation Facility, 71 Avenue des Martyrs, 38043 Grenoble, France; b Institute of Resource Ecology, Helmholtz Zentrum Dresden Rossendorf, Bautzner Landstrasse 400, 01328 Dresden, Germany; c Department of Research Technology, Helmholtz Zentrum Dresden Rossendorf, Bautzner Landstrasse 400, 01328 Dresden, Germany; d Institut für Experimentelle Physik, TU Bergakademie Freiberg, 09596 Freiberg, Germany; e European Synchrotron Radiation Facility, 71 Avenue des Martyrs, 38043 Grenoble, France; f Swiss Norwegian Beamlines, European Synchrotron Radiation Facility, 71 Avenue des Martyrs, 38043 Grenoble, France

**Keywords:** actinides, EXAFS, XANES, HERFD-XANES, XAS, XES, RIXS, XRD, CTR, RAXR, surface diffraction

## Abstract

ROBL-II provides four different experimental stations to investigate actinide and other alpha- and beta-emitting radionuclides at the new EBS storage ring of ESRF within an energy range of 3 to 35 keV. The XAFS station consists of a highly automatized, high sample throughput installation in a glovebox, to measure EXAFS and conventional XANES of samples routinely at temperatures down to 10 K, and with a detection limit in the sub-p.p.m. range. The XES station with its five bent-crystal analyzer, Johann-type setup with Rowland circles of 1.0 and 0.5 m radii provides high-energy resolution fluorescence detection (HERFD) for XANES, XES, and RIXS measurements, covering both actinide *L* and *M* edges together with other elements accessible in the 3 to 20 keV energy range. The six-circle heavy duty goniometer of XRD-1 is equipped for both high-resolution powder diffraction as well as surface-sensitive CTR and RAXR techniques. Single crystal diffraction, powder diffraction with high temporal resolution, as well as X-ray tomography experiments can be performed at a Pilatus 2M detector stage (XRD-2). Elaborate radioprotection features enable a safe and easy exchange of samples between the four different stations to allow the combination of several methods for an unprecedented level of information on radioactive samples for both fundamental and applied actinide and environmental research.

## Abstract 2

ROBL-II at ESRF provides four experimental stations to investigate actinides with X-ray absorption and emission spectroscopy, and with surface, high-resolution powder, and single-crystal X-ray diffractometry.

## 1. Introduction   

**Figure 1:**
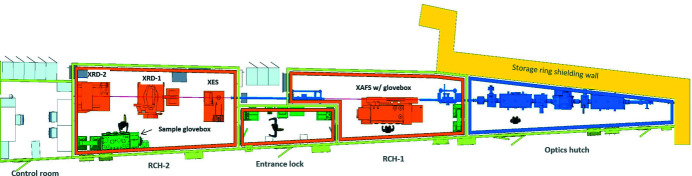
Layout of ROBL-II, showing from right to left the X-ray Optics hutch (blue), the two experimental hutches (RCH-1 and RCH-2) connected by a common entrance lock room (orange), and the control room. The X-ray beam marked by the red line enters from the right through a port in the shielding wall. RCH-1 houses the XAFS experiment, RCH-2 a five-crystal spectrometer (XES), a six-circle goniometer for powder and surface diffraction (XRD-1), and a Pilatus3 2M diffractometer (XRD-2). Hutch walls are shown in light green, radiation protection equipment in green.

The Rossendorf beamline has been operated at the ESRF since 1996 by Helmholtz-Zentrum Dresden-Rossendorf (HZDR) (Nitsche, 1995 ▸; Matz *et al.*, 1999 ▸; Reich *et al.*, 2000 ▸) and has been redesigned in 2016–2020 (ROBL-II). Embedded within the Helmholtz Association’s program on nuclear safety, ROBL-II is dedicated to research on actinides and other elements with no stable isotopes (Tc, Po, Ra). The beamline hosts four major experimental stations for synchrotron X-ray experiments in two radiochemistry hutches (RCH-1 and RCH-2) (Fig. 1 ▸):

(1) XAFS station with fluorescence and transmission detection for X-ray absorption fine-structure (XAFS) spectroscopy, including (conventional) X-ray absorption near-edge structure (XANES) and extended X-ray absorption fine-structure (EXAFS) spectroscopies.

(2) XES station with a five-crystal Johann-type spectrometer for high-energy-resolution fluorescence-detection X-ray absorption near-edge spectroscopy (HERFD-XANES), X-ray emission spectroscopy (XES) and resonant inelastic X-ray scattering (RIXS) measurements.

(3) XRD-1 station with a heavy-duty, Eulerian cradle, six-circle goniometer for (high-resolution) powder X-ray diffraction (PXRD), surface-sensitive crystal truncation rod (CTR) and resonant anomalous X-ray reflectivity (RAXR) measurements.

(4) XRD-2 station with a Pilatus3 x2M detector stage for single crystal X-ray diffraction (SCXRD) and *in situ*/*in operando* PXRD measurements.

## 2. Optics and control system   

**Figure 2:**
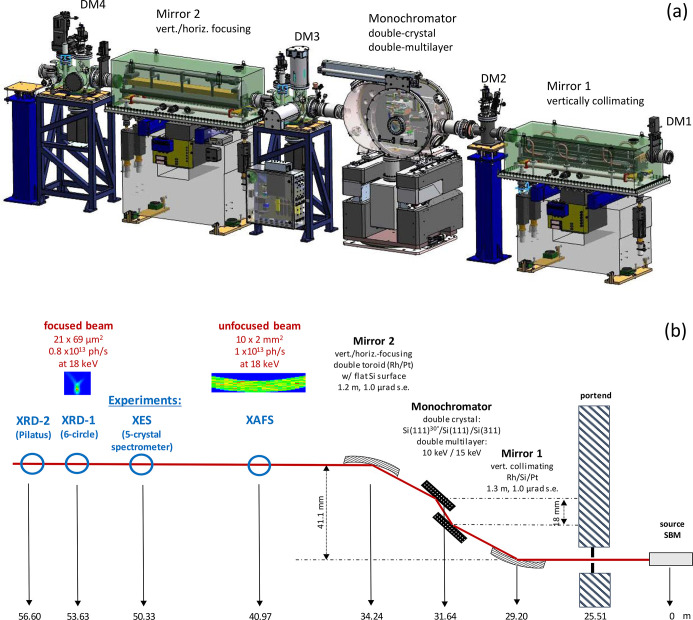
(*a*) Optical components provided by FMB Oxford and (*b*) schematic layout of the beam path (in red, from right to left) through optical components (black) to experimental stations (blue). The XAFS station receives only unfocused beam, while XES, XRD-1 and XRD-2 receive either focused beam from the Rh- or Pt-coated toroid sections of mirror 2, or (horizontally) unfocused beam from the flat Si section between the two toroids.

ROBL-II receives synchrotron beam from a short bending magnet (SBM) with 0.86 T of the new ESRF storage ring, a 7BA lattice operating at 200 mA and 6 GeV. This SBM provides ROBL-II with a spectral flux of 1.4 × 10^13^ photons s^−1^ (0.1% bandwidth)^−1^ at 18 keV, *i.e.* with about the same flux as the 0.85 T bending magnet of the previous storage ring of the ESRF, but with almost an order of magnitude higher brightness due to the smaller source size. The synchrotron beam is conditioned by the multipurpose optics (designed and delivered by FMB Oxford) shown in Fig. 2 ▸.

This optics setup consists of a combined double-crystal monochromator (DCM), and a double-multilayer monochromator (DMM). The DCM produces monochromatic X-rays within 3 to 35 keV with energy resolution of a few eV (depending on the employed crystal pair and energy) for spectroscopy at the expense of photon flux. The DMM is used to produce an about 100 times higher photon flux in the energy range 8 to 19 keV at the expense of energy resolution (∼100 eV) for scattering and selected spectroscopy applications, where energy resolution is not as critical or is improved by secondary monochromatization, *e.g.* using the five-crystal spectrometer of the XES station. Two mirrors before and after the monochromator collimate and focus the beam, respectively, and suppress higher harmonics. Diagnostic modules (DM) consisting of (removable) fluorescence screens and/or photon-sensitive movable blades and diodes are installed after the portend and after each optic element. Fig. 2 ▸ (bottom) shows also the final footprint of photons for the two groups of experimental stations, a large, unfocused beam for XAFS, and a small, focused beam for the other three stations, as calculated by ray tracing (*Shadow-Vui,* Oasys Version 1.0) and verified by computationally more expensive wavefront propagation methods. A more detailed description of the optics components is given in the following.

### 2.1. Monochromator   

**Figure 3:**
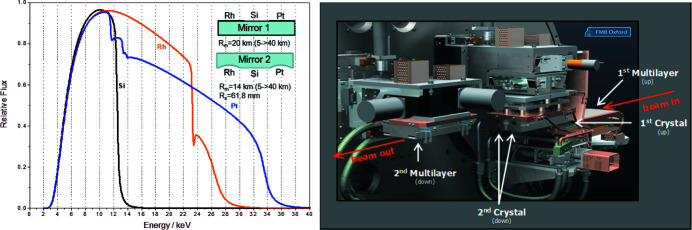
Left: reflectivity of the Si, Rh and Pt surfaces of mirrors 1 and 2, calculated with *XOP* (https://beam.aps.anl.gov/apps/xop/) for 2.5 mrad slope, 0.5 nm surface roughness and 1.1 mm Be. Right: design detail of the DCM/DML monochromator setup.

This unique design (FMB Oxford) combines a double-crystal (DCM) setup with three laterally mounted sets of crystals (which can be exchanged by laterally shifting the UHV vessel on the granite support), with a double-multilayer (DMM) setup with two laterally mounted sets of multilayers (which can be again exchanged by laterally shifting the UHV vessel), which can be exchanged without breaking the vacuum. The upward-bouncing, liquid-notrogen-cooled first crystals, as well as the upward-bouncing, water-cooled first multilayer crystals, are mounted on a common Bragg axis. The exchange from the DCM to the DMM setup is simply effectuated by moving the monochromator vessel upward until the multilayer crystals are in the beam (Fig. 3 ▸, right). The DCM setup comprises two Si(111) pairs, with a 30° difference in the crystal orientation towards the incoming beam, for an improved management of so-called glitches, caused by *Umweganregung*, especially in the energy region of actinide *L*_III_-edges (16 to 20 keV). A third crystal pair uses the improved energy resolution of the Si(311) plane. The DMM setup consists of two pairs of multilayers (ML1 and ML2, produced by AXO), with ML1 covering the energy range 8.5 to 12.6 keV (optimized for 10 keV), and ML2 from 12.6 to 18.7 keV (optimized for 15 keV). The diffracting optics consists of a silicon substrate coated with Mo/B_4_C multilayers of two different period thicknesses plus a ∼4 nm Si cover layer for protection, which can be rotated within an angle of 0.76 to 1.13° and provide an energy resolution Δ*E*/*E* < 1%. ML1 has 190 layers with a period thickness of 2.5 nm providing a reflectivity *R* of 0.9%; ML2 has 80 layers with a period thickness of 3.7 nm and an *R* of 2.3%.

### 2.2. Mirror 1 (collimating)   

The first, 1.3-m long, water-cooled mirror (Thales SESO) is set at an upward reflecting angle of 2.5 mrad and longitudinally bent to ∼20 km to collimate the white beam vertically. The silicon body is coated with two 37 mm-wide stripes of Rh and Pt, with an uncoated bare Si surface of 32 mm width in between (Fig. 3 ▸, left). The three different reflecting surfaces have been selected to maximize the flux across the working energy range (3 to 35 keV), while minimizing the contribution of higher-order harmonics (Fig. 3 ▸, left). Surface metrology performed by the ESRF metrology laboratory showed a surface (micro-) roughness of 0.4 nm for the 700 Å Rh and Pt coatings, and 0.6 nm for the bare Si surface. Slope errors (r.m.s.) vary between 0.7 and 1.1 µrad depending on stripe and bending radius. The meridional bending acts with excellent linearity between radius and motor steps (*R* ^2^ > 0.996) with no significant hysteresis between increasing and decreasing the radius.

### 2.3. Mirror 2 (focusing)   

The 1.2 m long, downward-reflecting mirror of monocrystalline silicon (Thales SESO) has two toroidal grooves both with a radius of 61.8 mm, one coated with Rh and the other with Pt, to focus the beam both horizontally and vertically into the XES, XRD-1 and XRD-2 stations with a spot size of about 20 µm × 70 µm (h × v), while the XAFS station is intentionally out of focus to minimize beam damage of samples (beam size on sample up to 10 mm × 2 mm, see Figs. 2 ▸ and 3 ▸). The 38 mm-wide flat section between both toroidal grooves is uncoated, *i.e.* the bare Si surface can be used to provide a collimated beam of <14 keV for diffraction experiments, where a beam with minimized divergence is required. Surface metrology provided surface (micro-) roughnesses of 0.3 nm for the 600 Å Rh coating and the bare Si surface, while the 1.3 nm for the 600 Å Pt coating is less favorable. Slope errors (r.m.s.) are 0.5, 1.0 and 1.3 µrad for Rh, Si and Pt surfaces, the value slightly above specifications for Pt most likely correlated to the high micro-roughness. Meridional bending behavior measured between 5 and 20 km was as favorable as for mirror 1.

### 2.4. Diagnostic modules   

Diagnostic and beam-defining modules (FMB Oxford) are installed after the portend (DM 1) and after each optical element (DM 2 to DM 4) (Fig. 2 ▸, top). Vertically and horizontally defining slits, consisting of four movable tungsten blades, are installed in DM 1, 2 and 3, and as far as they receive white/pink beam, they are water-cooled (DM 1 and 2).

Fluorescent screens are installed in DM 2, 3 and 4. They consist of a 25 µm-thick Y_2_O_3_ coating on a water-cooled oxygen-free high-thermal-conductivity copper (OFHC) block in the case of DM 2, and of a 50 µm-thick Y_2_O_3_ coating on an (uncooled) aluminium block in the case of DM 3 and 4. They are introduced into the beam via pneumatic actuators, and are remotely viewed by CCD cameras.

Beam position monitors are installed in DM 1, 2 and 4. In DM 1, a blade beam position is installed for permanent position monitoring of the white beam, consisting of four graphite blades mounted onto two water-cooled OFHC copper blocks. In DM 2, the four slit blades have voltage taps to measure drain current to enable the slits to be used as beam monitors. In DM 4, a profile monitor allows to map the beam vertically and laterally with high resolution, using GaAsP photodiodes (Schottky-type) behind two pinholes of 100 and 300 µm diameter.

### 2.5. Control system   

All motorized components of the optics as well as of the experimental stations described below are driven by IcePAP motor controllers, an ESRF inhouse development (http://www.esrf.eu/Instrumentation/DetectorsAndElectronics/icepap), except for the monochromator unit, where Pmac controllers are used (Delta Tau Data Systems Inc.). The main experiment control system is currently migrated from SPEC (Certified Scientific Software) to BLISS (BeamLine Instrumentation Support Software), an inhouse development of ESRF (https://www.esrf.eu/fr/home/UsersAndScience/support-and-infrastructure/software/bliss---beamline-control-software.html). It comes with a Python-based command line interface, a new scanning engine, a web-based configuration interface, and an online (live) and offline data visualization graphical interface. Fast switching between different optical configurations, including different mirror coatings and shapes and different DCM/DMM modes, can be achieved by a macro, which reads stored motor positions for different optical configurations from a table, and then moves all motor axes incrementally to the selected values. Note that all motors are equipped with encoders to obtain highly reproducible positions.

ROBL-II will also implement the ESRF data policy, which means that all data and metadata collected during an experiment will be stored onsite, searchable and accessible online (with a renewable embargo period of three years, then made public). Details on the data policy can be found here (https://www.esrf.eu/datapolicy).

## 3. Radiochemical safety system   

### 3.1. Common principles   

In order to be able to investigate actinoids and other elements with no stable isotopes (Tc, Po, Ra) at the ESRF, the following basic rules have been established (Funke *et al.*, 2001 ▸):

The radioprotection design is optimized for prevalently alpha- and beta-emitting radioisotopes; hence, for each of the above given elements, priority is given to the radioisotopes with the longest half-lifes. Exposure levels outside of the glovebox are limited to <0.5 µSv h^−1^, in contact with the sample enclosure <15 µSv h^−1^.

For all samples, which remain inside an underpressurized glovebox during measurements (currently only the XAFS station), an upper activity limit of 185 MBq is valid (Table 1 ▸).

Experiments can be conducted with three different types of samples: RAD-0 are non-active samples, which do not require any radioprotection treatment. RAD-1 are samples with activities below the threshold limits established for ESRF beamlines without dedicated radiochemistry equipment, which are *e.g.* 3.7 MBq for a group 1 radionuclide in solid form, less for liquids or powders (https://www.esrf.eu/Infrastructure/Safety/Experiments/RadioactiveSamples). RAD-2 are samples with activities below the 185 MBq limit established for ROBL. RAD-2 experiments require that samples are constantly maintained during the measurement in an underpressurized glovebox with ventilation, filter and radiation monitoring in place.

Otherwise, common principles for the construction and operation of ROBL-II are the multi-barrier containment for the samples, redundancy and automation of safety installations as well as automation of sample exchanges and measurements.

The hutches RCH-1 and RCH-2 do not only act as radiation shields against the synchrotron radiation (walls, doors, and roof covered by Pb shielding) but are in addition equipped as alpha-radionuclide laboratories by fulfilling specific requirements with respect to tightness and ease of decontamination (see Section 3.2 below). They are both accessed through an entrance lock with hand and foot monitors and body decontamination equipment, which serves also for a safe and easy sample transfer between the experimental stations in RCH-1 and RCH-2.

RCH-1 and RCH-2 are furthermore equipped with a redundant ventilation and filter system to establish a pressure gradient from the outside experimental hall of the ESRF to the hutches and then to gloveboxes in order to confine radionuclides in case of an accidental release from sample holders as close as possible to the source point (see Section 3.3 below).

RCH-1 and RCH-2 are finally equipped with a multistep radiation monitoring system and a radiochemical signal system (RCSS), which registers and stores the data provided by the radiation monitors and ventilation (air flux, pressure, temperature). RCSS automatically creates status reports, warnings and alarms in case of equipment failures and alpha/beta/gamma readings above the threshold, which are displayed at the beamline, in ROBL’s main office building, in the office of the hall operator and in the ESRF’s main control room (see Section 3.4 below).

RAD-2 experiments that require that samples are constantly maintained during the measurement in an underpressurized glovebox can currently only be performed in the XAFS station, where bulk EXAFS measurements commonly require sample masses and activity levels exceeding those of RAD-1. In contrast, experiments at XES, XRD-1 and XRD-2 can commonly be conducted with a microfocused beam and samples can hence be kept small enough to maintain the activity levels of RAD-1. Therefore, there are currently no gloveboxes installed around these three experiments. For all tasks associated with active sample reception, control and shipment, there is a dedicated glovebox available in RCH-2 with gamma-spectrometer and contamination monitors. Sample confinements and handling procedures are further detailed below.

The XAFS station in RCH-1 benefits in addition from a high degree of automation for sample exchange and positioning in the beam under both room-temperature and cryogenic conditions.

### 3.2. Experimental hutches   

The hutches RCH-1 and RCH-2 (Innospec) are part of the multi-barrier design concept, separating the hutch airspace from the experimental hall of the ESRF. As such, the walls and roof, as well as all openings like doors, windows (including alignment windows) and chicanes for ventilation, fluids and electrical cabling, do not only form a shielding for the synchrotron radiation but are also sufficiently air tight to maintain an underpressure of 50 to 100 Pa against the outside (leakage rate less than 1% of respective hutch volume). This is obtained by covering the walls and the roof with 1 mm-thick steel panels as inner lining, which are sealed by silicon or self-adhesive sealing tape. The removable roof elements and the doors are air-tightened with self-sealing rubber gaskets. The tightness of the fluid and cabling chicanes is realized by RoxTec (certified air and water tightness and fire resistance) (https://www.roxtec.com). The panels are polyester-coated for easy decontamination. All equipment inside the hutches is placed on 25 mm-thick aluminium or stainless steel plates. The remaining floor area is covered by an Ep­oxy liner, with air- and water-tight sealing against the floor plates and the wall lining.

### 3.3. Ventilation system   

**Figure 4:**
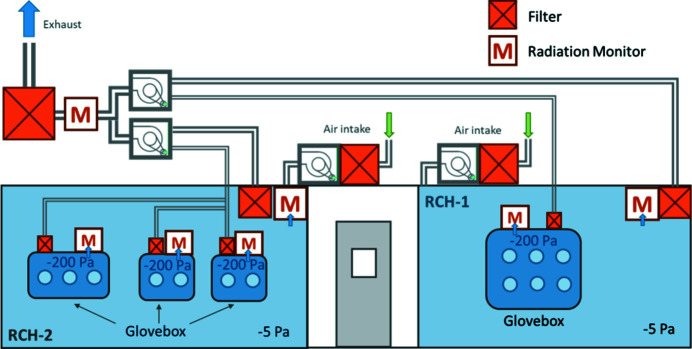
Radioprotection ventilation scheme. Note that only a simplified system is shown, *i.e.* all ventilators and all box and hutch filters are installed redundantly to ensure safety even in case of a failure of one of the components. The three gloveboxes shown in RCH-2 are optional.

The ventilation system (see schematic view in Fig. 4 ▸) is designed to protect personnel and users working at the beamline, at the ESRF and on the EPN campus from a potential radioactive contamination, caused by a failure of the various sample enclosures during sample handling and measurements. The ventilation system has therefore several tasks: (1) Produce a pressure gradient from the experimental hall (0 Pa) to the interior of the two hutches RCH-1 and RCH-2 (−5 Pa) and then to the gloveboxes (−200 Pa), in order to confine a potential contamination first to the gloveboxes and then to the hutches. (2) Keep the exhaust air contamination-free, first by filters at the exit of the gloveboxes in order to prevent a spreading of the contamination as close as possible to the source, second by filters at the exit of the hutches, and third by a filter at the combined exhaust in order to prevent a release of radioactivity into the environment. (3) Monitor a potential release of radioactive contamination inside the gloveboxes, *i.e.* as close as possible to the samples, inside the hutches to protect staff and users during sample exchanges, and finally before the total filter at the common exhaust of both hutches. (4) The two separate air intake units for RCH-1 and RCH-2 filter and temperature-condition the incoming air; that of RCH-2 also dries the incoming air. (5) The ventilation system is redundant: in case of a failure of one of the ventilators or of one of the glovebox and hutch filters, the system switches automatically to a parallel ventilator and filter set in order to maintain the pressure gradients and the safety of users. (6) The ventilation system is connected to an uninterruptable power supply (UPS) in order to ensure that the ventilation works even after a power outage for at least 30 min. (7) In case of fire inside one of the hutches, air intake and exhaust valves are immediately closed by automatic fire flaps. (8) To maintain the pressure gradient between hall and hutches, only one of the three doors, connecting hall and entrance lock, entrance lock and RCH-1, and entrance lock and RCH-2, can be opened at the same time. (9) Inside the hutches, the air is exchanged within the recommended bracket of 6 to 15 changes per hour, namely 750 m^3^ h^−1^ for RCH-1 and 1050 m^3^ h^−1^ for RCH-2. This is controlled by ultrasonic air flow meters in the exhaust lines.

Fig. 4 ▸ shows a simplified scheme of the ventilation systems, the filters and the radiation monitors, *e.g.* without redundant components.

A UPS provides power to the ventilation system long enough to enable personnel to leave RCH-1 and RCH-2 and to shut down the ventilation system in a controlled way. After a fault of the electrical power network, the UPS will first continue the operation of the ventilation system without the very power-consuming heaters of the air intake CTAs (RCH1 and RCH2). After the temperature of the heater unit has been sufficiently reduced by the ventilated air flow, the CTA ventilators will be stopped and the bypass of the air intake will be opened. The air flow will pass through the CTA without any ventilator running, and the underpressure in RCH1, RCH2 and the gloveboxes will be maintained solely by the air extractors located in the technical room (air extractor of the RCH1, RCH2 rooms, gloveboxes). About 45 minutes after the start of the UPS, the extractors will be stopped and all valves of the extraction network of the hutches will be closed to isolate the hutches from the outside.

### 3.4. Radiochemical signal system (RCSS)   

**Figure 5:**
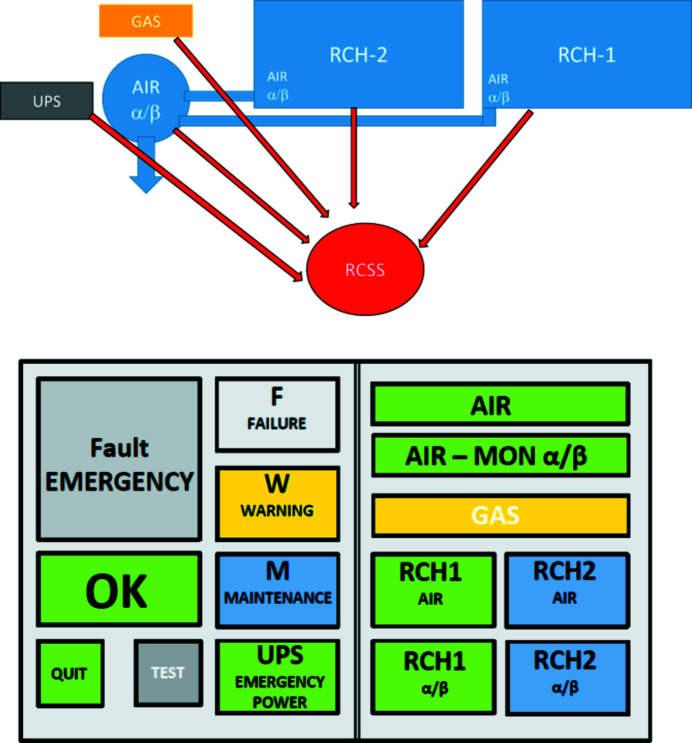
Radiochemical signal system (RCSS). Top: RCSS data collection (red) from ventilation (blue), counting gas (yellow), and UPS (gray). Bottom: RCSS display panel using gray colors for deactivated states, green for normal operating condition, yellow for warning, orange for failures and red for emergency status.

RCSS supervises the correct functioning of the ventilation system including the integrated monitors and – in case of an eventual failure – produces adequate signals to indicate the kind and seriousness of the failure. In detail, it takes care of the following tasks: (1) Collecting and analyzing the status data from all monitoring systems (normal, maintenance, warning, failure, emergency) (Fig. 5 ▸, top). (2) Displaying these status signals in the experimental hutches and the control room of ROBL as well as in the control room of the ESRF (Fig. 5 ▸, bottom). (3) Logging and storing the data in order to visualize trends for maintenance purposes.

The following readings are displayed by RCSS (Fig. 5 ▸, bottom). AIR: final air exhaust monitoring system. AIR-MON α/β: radiation monitoring system. GAS: P10 counting gas supply of the AIR-MON α/β monitor. Havary, Failure, Warning, Maintenance, OK, and UPS Emergency Power: system status. TEST: system test button. QUIT: quit alarm sound button.

In the *deactivated state* all the displays are shown in gray. When *activated*, the display fields are displayed in the colors blue (for maintenance), green (for ok), yellow (for warning), orange (for failure), and red (for emergency). In the example shown in Fig. 5 ▸, RCH-1 is in green, *i.e.* OK condition, RCH-2 is in blue, *i.e.* maintenance condition. The combined exhaust ventilation system is running ok, but the P10 counting gas symbol (GAS) shows a warning (low gas pressure).

Signal changes from green to yellow or orange are accompanied by a short signal tone, those to the red level by a continuous signal tone, which can be stopped only by pushing the QUIT button for at least 3 s. When a system is in MAINTENANCE status, the acoustic signals are suppressed for this system. One can use the TEST button to perform a signal and display test on the respective tablet. During this test, the acoustic signal is activated when the button is actuated and all display areas are displayed in gray.

### 3.5. Radioprotection procedures during experiments   

**Figure 6:**
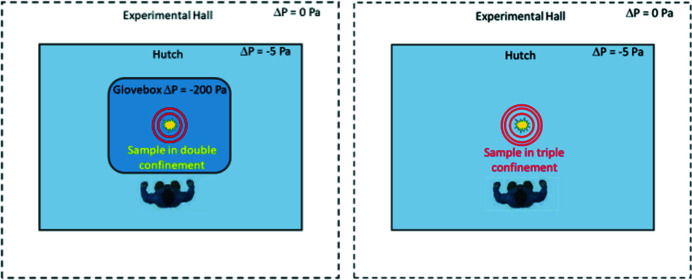
Radioprotection modes during experiments with (left) or without (right) an underpressurized glovebox.

For the four experiments established inside these two hutches, there are two different operation modes foreseen, either with or without glovebox. In the first mode, the samples are confined by a double-wall container and remain during the experiment inside an underpressurized (−200 Pa) glovebox (Fig. 6 ▸, left). In this mode, the full sample inventory of up to 185 MBq can be measured. In the second mode, the samples are confined by a triple-wall container and can be measured without glovebox (Fig. 6 ▸, right). In this mode, samples must remain within the radioactivity limits set up for other beamlines at the ESRF (*e.g.* 3.7 MBq for a group 1 radionuclide in solid form, less for liquids or powders).

A range of double- and triple-confinement sample holders certified by the ESRF Radioprotection Group and the corresponding HZDR radiation protection officer are made available to users. These account for different sample states (liquids, solids, wet pastes), temperatures (cryostat or room temperature) and techniques.

Each experiment is assigned to one of three risk groups, green for no risk, yellow for moderately radioactive samples (typically Tc, Th, U), and red for samples with substantial radiotoxicity (typically transuranium elements). For red experiments, requirements include the 24 h presence of one user at the beamline, and the presence of two persons, one of them a ROBL staff member, for sample exchanges. At the end of each radiochemistry experiment, the samples and experimental stations are checked for contaminations by the ESRF Radioprotection Group, and eventually decontaminated to ensure a contamination-free environment for the following experiments. The RCSS system ensures that ROBL-II users and staff as well as ESRF hall operators are always informed about the correct functioning of all radioprotection systems, and are warned in case of failures or contaminations by the RCSS systems, giving three levels from warning to failure to emergency (Fig. 5 ▸).

## 4. XAFS   

X-ray absorption fine-structure (XAFS) spectroscopy, including (conventional) X-ray absorption near-edge structure (XANES) and extended X-ray absorption fine-structure (EXAFS) spectroscopies, has been since the early 1990s one of the most universal methods to study actinide speciation in liquids, solids and at interfaces, with applications including fundamental, coordination, environmental and materials chemistry of actinides (Nitsche, 1995 ▸; Denecke, 2006 ▸; Geckeis *et al.*, 2013 ▸; Maher *et al.*, 2013 ▸; Epifano *et al.*, 2019 ▸). In fact, the first version of ROBL built in 1997 was dedicated to XAFS as a sole method to study actinide chemistry (Reich *et al.*, 2000 ▸). While additional methods are now provided as detailed in the following sections, XAFS will remain a fundamental method, while we expand its limits by novel technology. Premises for the new XAFS station were: (1) keep a relatively large beam footprint on the sample to minimize beam damage of sensitive samples (containing redox sensitive elements as well as water and organics with a tendency to produce radicals) during the often long exposure times of several hours; (2) keep the samples during measurements enclosed in an alpha-glovebox in order to profit of the higher activity limit (185 MBq, RAD-2, see Section 3 above); (3) provide routinely measurements in a closed-cycle He cryostat to block chemical reactions possibly induced by time, exposure to atmospheric oxygen and high photon flux; (4) minimize user intervention by using automatized sample changes not only for room temperature but also for cryogenic measurements; (5) shift the lower detection limit as far as much into the sub-p.p.m. concentration range as is currently achievable.

**Figure 7:**
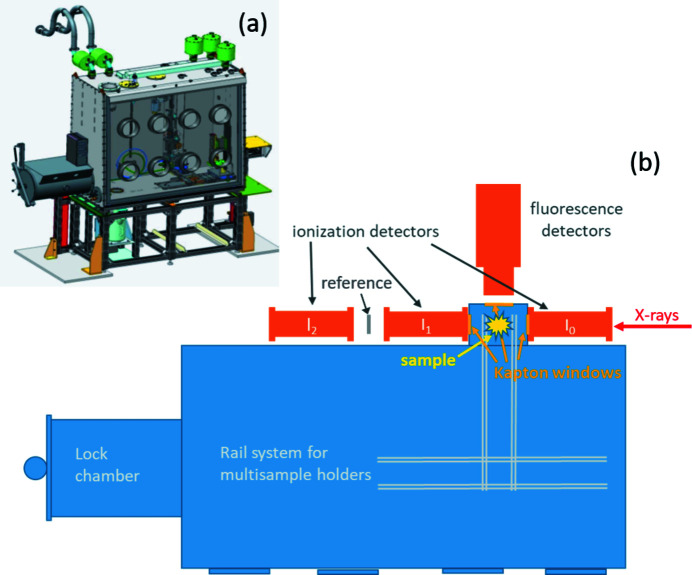
XAFS glovebox. (*a*) Design and (*b*) conceptual drawing (top view).

A large (2 mm × 10 mm) beam is obtained by placing the XAFS station far out of focus of the toroidal mirror (Fig. 2 ▸). A glovebox has been designed, where the samples are exposed to the monochromatic synchrotron beam passing through a back-side extension with Kapton windows, while ionization and fluorescence detectors are at the outside (Fig. 7 ▸).

**Figure 8:**
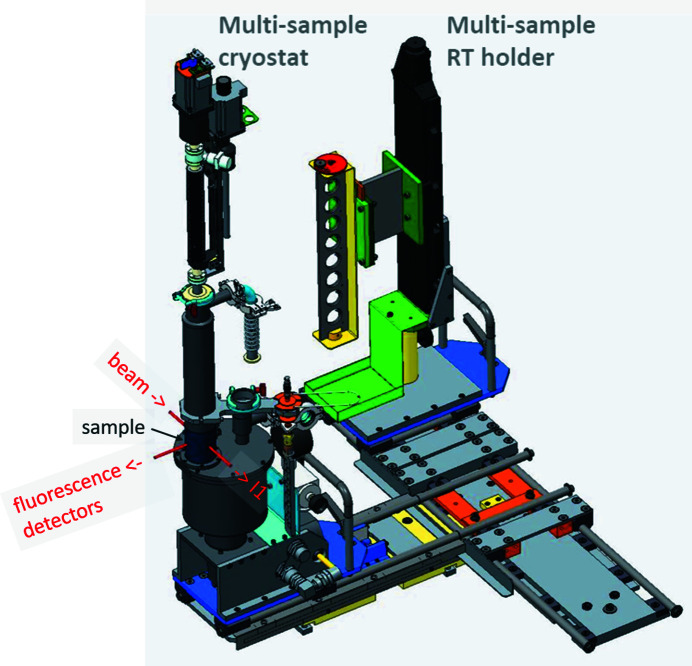
Motorized multi-sample stages for cryogenic and room temperature (RT) measurements in the XAFS glovebox. The multi-sample cryostat is shown in measurement position, *i.e.* moved into the backside extension of the XAFS glovebox [see sample position in Fig. 7 ▸(*b*)], while the RT holder is in park position. The cryostat is shown fully extended (height 1260 mm), *i.e.* with the lowest sample in the beam.

The glovebox contains two automated sample stages, one for room-temperature (RT) XAFS measurements (multi-sample RT holder) and one for cryogenic EXAFS measurements (multi-sample cryostat) (Fig. 8 ▸). The RT stage consists of a holder for up to eight samples, which can be moved vertically by a motor to bring each sample into the incident beam. The cryogenic sample stage consists of a custom-made closed-cycle He cryostat (CryoVac). Similar to the RT stage, a motorized vertical translation moves a six-sample stage inside the cryostat in order to bring the individual samples into the incident beam. Each of the two stages can be moved manually on a tray system between a front position for sample loading, and the back position for the measurement.

Finally, two electrically cooled, Ge or Si multi-element solid state detectors are available, with individual elements and CMOS-based charge-sensitive amplifiers named CUBE (XGLab) for increased count-rate throughput, and Falcon-X read-out spectrometers (XIA). The first one is an electrically cooled (Cryo-Pulse 5 Plus) 18-element germanium detector (Ultra-LEGe, GUL0055, Mirion Technologies) with 50 µm-thick elements providing a spectral resolution of ∼150 eV at 100 kcounts s^−1^. The gain is ∼3.9 mV keV^−1^, reset time <1 µs, and rise time <40 ns. The second one is a seven-element silicon DRIFT detector (SDD) with 500 µm-thick Si elements and equipped with a 1.5 W pulse tube cooler (PIPS model SXD7X50M-500-CM-CC_01, Mirion Technologies). The best spectral resolution is <130 eV, which raises to <160 eV at 1 Mcount s^−1^. While the Si detector has a higher performance at energies below 12 keV and the Ge detector at energies above, the Si detector nozzle is arranged off-center so that both nozzles can be mounted as close as possible to each other for simultaneous use. Due to their advanced (CUBE-CMOS) electronics and fast readout electronics (XIA Falcon-X), both detectors can handle about 10^6^ counts element^−1^ s^−1^ with reasonable dead-time (<30%), and are hence able to collect EXAFS spectra of samples with concentrations below 1 p.p.m. in reasonable time.

**Figure 9:**
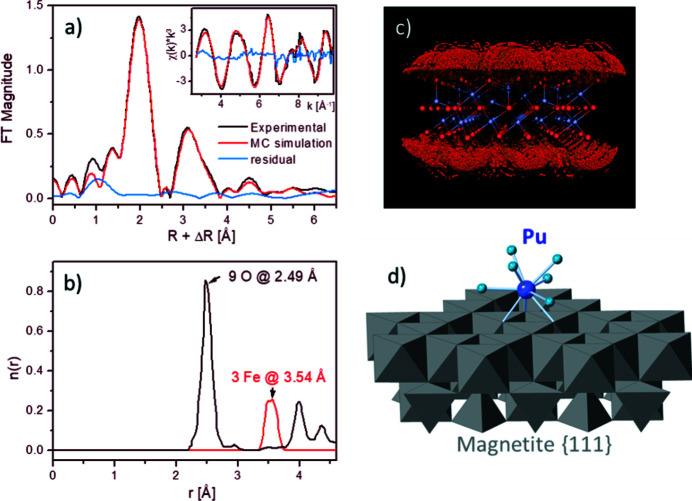
Advanced EXAFS analysis of Pu(III) sorbed at magnetite. (*a*) Experimental Pu *L*_3_-edge EXAFS spectra (black) shown as *k* ^3^-weighted chi function (insert) and its Fourier transform magnitude with Monte Carlo (MC) simulation in red. (*b*) Pu–O (black) and Pu–Fe (red) pair distribution function derived from the EXAFS data in (*a*) by MC fit. (*c*) Slab of magnetite cut along the {111} face (Fe in blue balls, O in red balls). The orange to green balls near the upper and lower magnetite faces depict MC-derived Pu positions with decreasing error between the experimental and MC simulated EXAFS spectra shown in (*a*). (*d*) Position of the Pu^III^ nona-aqua cation at the magnetite surface derived by MC simulation (Kirsch *et al.*, 2011 ▸).

The high EXAFS performance on the detector side is supplemented by a unique set of inhouse data analysis tools, like wavelet plot analysis using Cauchy or FEFF-derived wavelets (Funke *et al.*, 2005 ▸, 2007 ▸), iterative transformation factor analysis (ITFA) (Rossberg *et al.*, 2003 ▸, 2009 ▸; Yalçıntaş *et al.*, 2016 ▸), also coupled with Monte Carlo (MC) simulations (Rossberg & Scheinost, 2005 ▸; Kirsch *et al.*, 2011 ▸), the direct derivation of the radial pair distribution by Landweber iteration (Rossberg & Funke, 2010 ▸), and artificial intelligence approaches (Domaschke *et al.*, 2014 ▸), in order to derive as much information as possible from this versatile technique. Fig. 9 ▸ shows as flagship example the derivation of the structure of Pu(III)-sorbed to magnetite by MC simulations of the Pu EXAFS signal at the magnetite surface [Figs. 9(*a*) and 9(*c*) ▸], resulting in the pair distribution function [Fig. 9(*b*) ▸] and the refined structure of the highly specific, triple-edge sharing surface complex at the oxygen-terminated magnetite {111} face.

## 5. XES   

**Figure 10:**
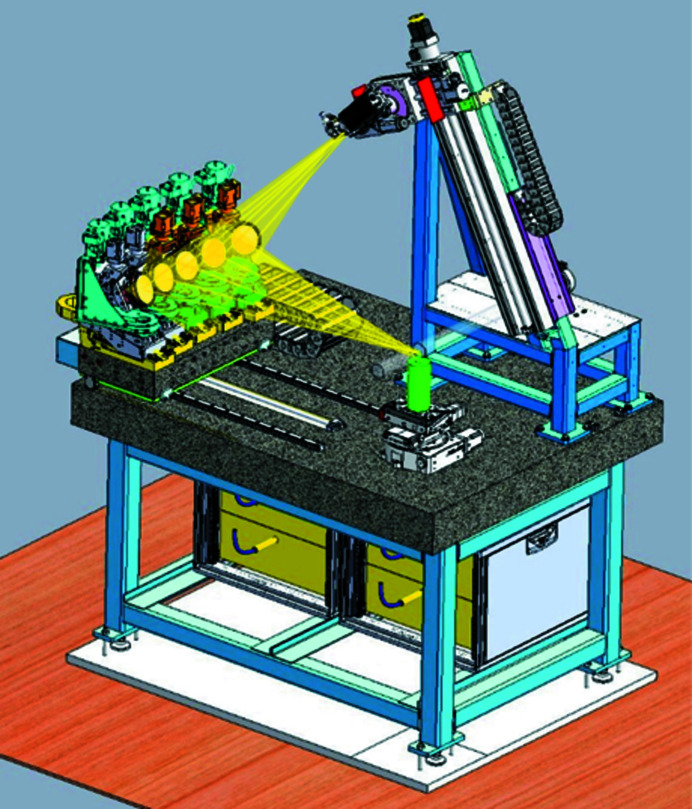
Schematic drawing of the five-crystal X-ray emission spectrometer.

X-ray emission spectrometers are used for several techniques, including X-ray emission spectroscopy (XES), high-energy-resolution fluorescence-detection X-ray absorption near-edge structure spectroscopy (HERFD-XANES), resonant inelastic X-ray scattering (RIXS) and/or resonant inelastic X-ray emission spectroscopy (RXES) (De Groot & Kotani, 2008 ▸; Van Bokhoven & Lamberti, 2016 ▸; Glatzel & Bergmann, 2005 ▸). Two spectrometers are available at ROBL: a one-crystal and a five-crystal spectrometer. The one-crystal spectrometer is used only for specific experimental arrangements, which require detection of emitted X-rays in particular scattering geometries (Kvashnina & Scheinost, 2016 ▸). The five-crystal spectrometer is the principal instrumentation for standard HERFD-XANES, XES, RIXS and RXES experiments (Fig. 10 ▸). The spherically bent crystal analyzers, the sample and the detector are mounted in vertical Rowland geometry. Each of the five crystals monochromatizes the fluorescence according to Bragg’s law and focuses the reflected monochromatic photons into the detector (Kleymenov *et al.*, 2011 ▸; Hazemann *et al.*, 2009 ▸; Huotari *et al.*, 2006 ▸; Sokaras *et al.*, 2013 ▸; Rovezzi *et al.*, 2020 ▸; Kavčič *et al.*, 2012 ▸). The resolution of the instrument depends on the selected Bragg angle of the analyzer and is thus linked to the selected energy. The best resolution is obtained in the backscattering geometry at Bragg angles close to 90° due to the geometrical effect of the Johann geometry (Kleymenov *et al.*, 2011 ▸; Sokaras *et al.*, 2013 ▸). The resolution is further improved by a motorized slit (typically 1 mm) in front of the detector. ROBL’s five-crystal spectrometer covers the Bragg angles 65–89°. For concentrated samples an avalanche photodiode (APD) with an area of 10 mm × 10 mm, and for dilute samples a Ketek detector with 10 mm diameter and 450 µm Si thickness are available.

The five-crystal spectrometer can be operated with crystal analyzers with 0.5 m and 1 m bending radius (Rovezzi *et al.*, 2017 ▸). Available spherically bent striped crystal analyzers (∼100 mm diameter) cover most elements of the periodic table in the energy range 3–20 keV (Table 2 ▸). Note that the whole spectrometer setup (sample, analyzer crystals and detector) can be placed into a He-filled bag to obtain sufficient fluorescence yield also at the lowest energies.

Energy scans are based on the Bragg angle and trigonometric equations that follow the Rowland circle geometry (Kvashnina & Scheinost, 2016 ▸). Each crystal analyzer is mounted on four motorized translation stages: two linear stages for the up/down and horizontal (closer or farther from the sample) motions, a goniometer to focus the fluorescence on to the detector, and a cradle for Bragg angle selection. The detector is mounted on two perpendicular, linear stages, and a goniometer to follow the Bragg angle reflection and to use different detector angles.

**Figure 11:**
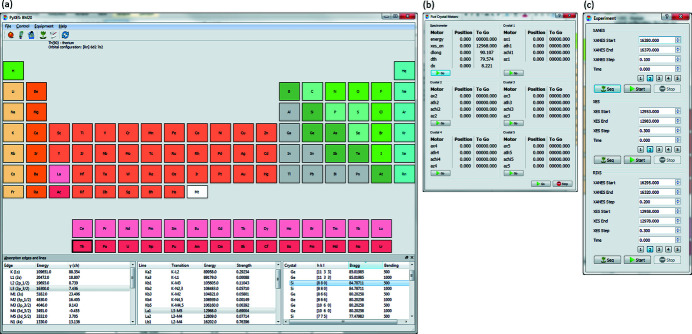
Screenshot of the custom-designed PyXES software. (*a*) Periodic table of the elements. After selection of the element and its absorption edge, the corresponding fluorescence lines and the appropriate analyzer crystal is given. (*b*) Calculation of motor positions corresponding to the chosen settings in (*a*). (*c*) Scan settings for XANES, XES, and/or RIXS measurements.

HERFD-XANES measurements are performed by scanning the incident energy across the absorption edge of the selected element at the maximum of the X-ray emission line. XES spectra are measured by scanning the emitted energy with the crystal analyzers, while keeping the incident energy fixed. If the incident energy is selected above the X-ray absorption edge, non-resonant XES is recorded. If the incident energy is selected below or near the absorption edge, RXES or/and RIXS is recorded. In order to minimize the down-time between the different techniques and to increase the user-friendliness of the spectrometer operation in general, the *PyXES* software has been developed, which allows to select the chemical element, the absorption edge (*K*, *L*, *M*), the emission line, and the crystal analyzer set, and then based on this input moves all motors positions into position (Fig. 11 ▸). Energy ranges for HERFD-XANES, XES, and RIXS are proposed automatically and can then be adjusted by the user. Additionally, batch scripts can be written in order to set up automatic measurements for several samples and/or techniques. The current position of all spectrometer motors and their expected position upon the selection of a particular experiment are displayed.

**Figure 12:**
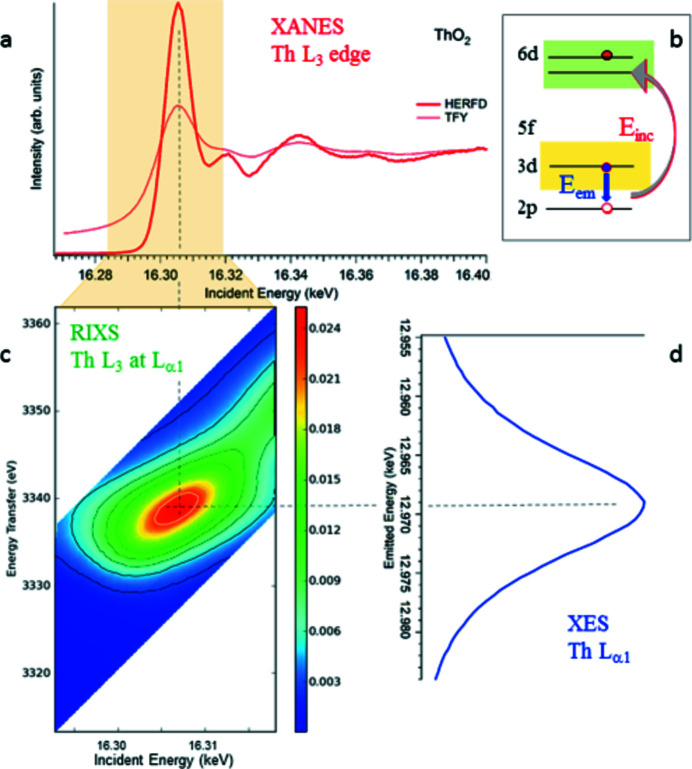
XANES, XES, and RIXS of ThO_2_. (*a*) Th *L*_3_-edge XANES spectra recorded in the conventional, total fluorescence yield (TFY) mode in comparison with the HERFD mode. (*b*) Electronic transitions of the XANES, XES, and RIXS process. (*c*) RIXS map recorded near the maximum of the Th *L*_α1_ emission line with incident energies near the maximum of the Th *L*_3_ edge. (*d*) XES Th *L*_α1_ spectrum recorded with non-resonant excitations.

We demonstrate here the different techniques using ThO_2_, prepared as a powder sample sealed between two layers of Kapton foil of 25 µm thickness (Amidani *et al.*, 2019 ▸). Fig. 12 ▸(*a*) shows the conventional Th *L*_3_-edge XANES (collected in normal, total fluorescence yield mode without crystal analyzer, ‘TFY’) in comparison with the HERFD-XANES spectrum recorded at the maximum of the Th *L*α_1_ (3*d*_5/2_–2*p*_/2_) emission line (∼12968 eV) as a function of the incident energy (collected in about 5 min). The emission energy was selected using the (880) reflections of five spherically bent striped Si crystal analyzers with 0.5 m bending radius aligned at 84.4° Bragg angle. The intensity was normalized to the incident flux. A combined (incident convoluted with emitted) energy resolution of 2.5 eV was determined by measuring the full width at half-maximum (FWHM) of the elastic peak of tungsten foil. The full RIXS map [Fig. 12 ▸ (*c*)] established by scanning the incident energy at different emission energies was recorded in about 30 min. The XES shown in Fig. 12 ▸(*d*) was obtained by scanning the X-ray emission energy at fixed incident energy above the Th *L*_3_ edge (∼16.5 keV). Following the dipole selection rules, the XANES spectrum arises from electron excitations from the ground 2*p* level to the unoccupied 6*d* level [Fig. 12 ▸(*b*)]. The core hole created by this process is very unstable and quickly filled in by an electron from the higher levels. The X-ray photons emitted during the process 3*d* → 2*p* are measured by XES at the Th *L*­_α1_ emission line.

## 6. XRD-1 (six-circle diffractometer)   

**Figure 13:**
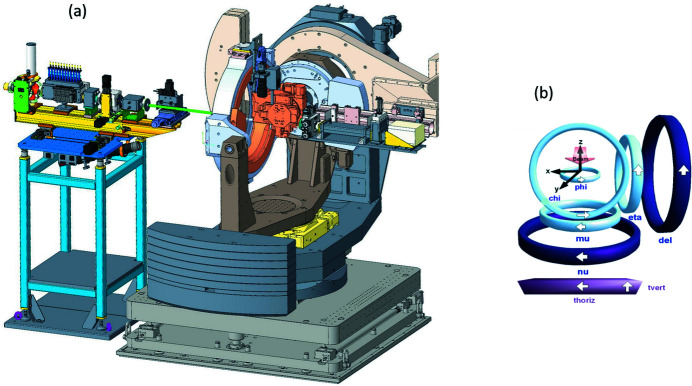
(*a*) Six-circle diffractometer (Huber Diffraktionstechnik GmbH) with the beam conditioning unit (left) and (*b*) geometrical arrangement of the goniometers and linear stages. Detector goniometers are shown in dark blue, sample goniometers in light blue.

The XRD-1 endstation with a six-circle diffractometer (Huber Diffraktionstechnik GmbH) is adapted to analyze well defined single reflexes with high resolution, and is hence suited for high-resolution powder diffraction as well as for surface diffraction. The diffractometer is equipped with a rather small detector mounted on a rigid detector arm, which can freely rotate in relation to the sample mount (Fig. 13 ▸). The diffraction angle 2θ is defined by the mechanical angle between the detector position and the primary beam. The construction is based on the classical Eulerian cradle geometry. The detector arm can be moved in the horizontal (nu) and in the vertical (del) scattering plane, as well as in any direction combing the horizontal and vertical movements (Fig. 13 ▸, right).

Two rotation axes of the sample goniometers (mu and eta) are aligned parallel to the rotation axes of the detector (nu and del) to allow a precise θ/2θ scattering geometry in any spatial direction. The Eulerian cradle with an inner diameter of 500 mm carries the sample support, which consists of an independent phi-rotation axis and an *x*/*y*/*z* table. The axis of the Eulerian cradle (chi) is aligned perpendicular to the two detector axes. The complete diffractometer can be moved horizontally (thoriz) and vertically (tvert) to place the rotation center in the geometrical focus of the X-ray beam. All rotational axes are equipped with encoders.

The incoming X-ray beam is shaped and monitored with the beam conditioning unit, which is placed in front of the diffractometer (Fig. 13 ▸, left). Its components are modular and easily exchangeable. The intensity of the incoming X-ray beam is counted by a monitor, which consists of a 45°-tilted, 20 µm Kapton scattering foil and an LaCl_3_ scintillation counter (FMB Oxford/Cyberstar). The second module allows to reduce the primary intensity by remote-controlled pneumatic insertion of different metal foils. A third module suppresses parasitic scattering with a vacuum-tight slit (IB-C22-HV, JJ-X-ray). The fourth module is a fast shutter (RI Research Instruments GmbH) to protect the final detector from too intense reflections during goniometer and detector movements. Module 5 is an ionization chamber (IC Plus 50, FMB Oxford) to monitor the true incoming X-ray intensity for normalization. Module 6 is a pinhole, which cuts off the remaining parasitic beam scattering off the tungsten blades of the slit. The unit is under vacuum except for the last two components.

**Figure 14:**
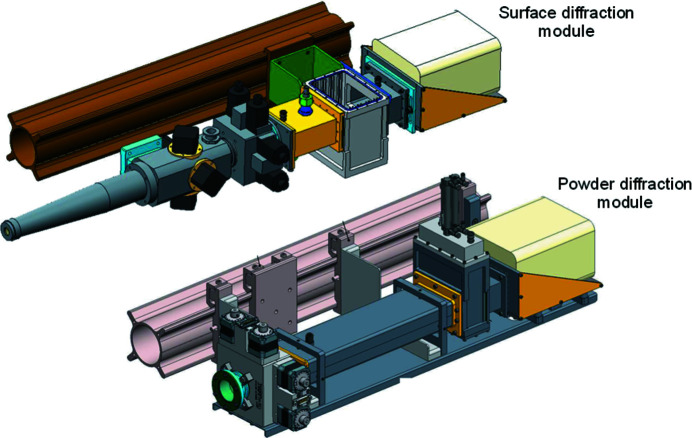
Detection modules of the six-circle diffractometer.

Two detection modules are available, one for surface diffraction and one for high-resolution powder diffraction (Fig. 14 ▸). Both modules use a Pilatus 100k detector with a 450 µm Si sensor (Dectris Ltd). The beam reflected from the sample hits the module for surface diffraction (Fig. 14 ▸, top) first through an in-vacuum tube slit (Huber Diffraktionstechnik GmbH) to reduce air scattering after the sample. Another vacuum-tight slit (IB-C22-HV, JJ-X-ray) allows further reduction of the scattering window. Then follows a purpose-built ionization chamber with 30 mm distance between the HV electrodes; this large distance is necessary to illuminate the whole detector area. This ionization chamber is used to align the sample in the direct beam. A pneumatic protection module with two 0.5 mm-thick tungsten blades closes automatically, when the detector arm moves close to the primary beam. It is controlled by SPEC-based software, which allows setting of the limits for closing the tungsten blades as well as deliberately opening them for detector alignment with intensity-reduced primary beam. The module for surface diffraction can be evacuated to <10^−3^ mbar. SPEC macros to operate the diffractometer for surface diffraction and for data registration have been provided by Peter Eng (GSECARS, 13 IDC, Advanced Photon Source). A long-distance microscope (K2, Infinity Photo-Optical GmbH) at 800 mm distance from the diffractometer center permits optical sample alignment.

While the highest resolution in powder diffraction can be achieved with secondary analyzer crystals (Dejoie *et al.*, 2018 ▸; Hodeau *et al.*, 1998 ▸), we refrained from this approach, since installation and alignement of the analyzer crystals turned out to be too time consuming for user operation with experiments changing typically from week to week. Instead, as a compromise between short installation time and reasonable resolution, we developed a module for the small Pilatus 100k detector also used for surface diffraction, with a sample–detector distance of 800 mm (Fig. 14 ▸, bottom). A detector with smaller pixel size will be procured in the future. The resolution of this setup depends on the used sample capillary diameter, which is typically 300 µm. The capillary is rotated with up to 200 r.p.m. The entrance slit for the powder diffraction module is a vacuum-tight IB-C30-HV model (JJ-X-ray), connected to an anti-scattering tube. The module contains also a pneumatic detector protection unit as described above. The entrance and the exit of the module is sealed by Kapton windows and can be evacuated to <10^−3^ mbar to reduce scattering from air.

**Figure 15:**
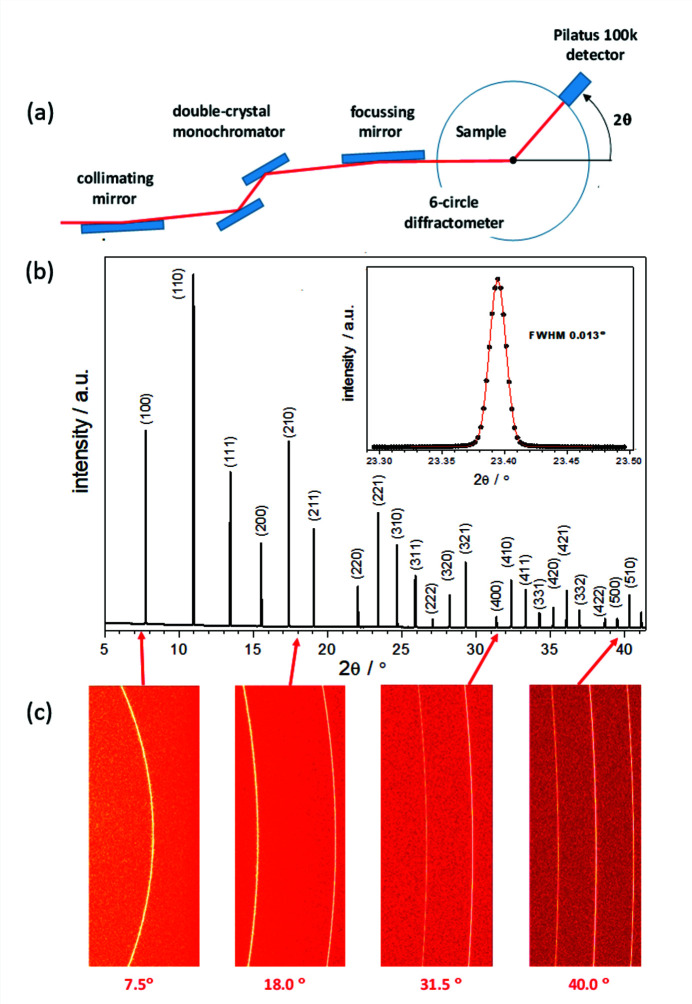
(*a*) Sketch of the setup for a high-resolution powder diffraction experiment with the Pilatus 100k detector. (*b*) High-resolution powder diffraction pattern of LaB_6_ with *E* = 220680.5 eV, λ = 0.5618 Å, glass capillary 0.3 mm diameter, sample–detector distance 800 mm, step width 0.5°. Insert: peak profil reflex (221) with a resolution Δ2θ of 0.013°. (*c*) Four of the 80 shots to obtain the full diffractogram shown in (*b*).

The Pilatus 100k detector has an active area of 33.5 mm × 83.8 mm (height × width) and contains 195 × 487 pixels with a size of 172 µm × 172 µm. A powder diffraction measurement is performed by a stepwise movement of the detector, *e.g.* with 80 overlapping shots at steps of 0.5° as shown for LaB_6_ (Fig. 15 ▸). The obtained pattern requires a radial integration along the intersections of the Debye–Scherrer cones with the detector surface, which can be done using the *pyFAI* software (Ashiotis *et al.*, 2015 ▸). This process includes corrections for small tilting errors of the detector. The detector calibration requires the measurement of standards (so-called calibrants). An independent determination of the wavelength is necessary due to its strong correlation with the sample–detector distance. As the detector surface of the Pilatus 100k is relatively small, the calibration needs to be done iteratively, starting with the measurement of the Debye–Scherrer cones at small 2θ angles using silver behenate [CH_3_(CH_2_)_20_COO·Ag] as first calibrant. Subsequently, the calibration is extended to detector positions at higher 2θ values using LaB_6_ as second calibrant. The extracted point of normal incidence (PONI) parameters are required for radial integration of the 2D powder pattern. The PONI parameters are: the sample–detector distance (dist), two orthogonal *x*- and *y*-dimensions in the detector plane (poni1, poni2), and three rotation parameters around the vertical axis (rot1), around the horizontal axis (rot2) and around the incoming beam (rot3). The calibration procedure is described in more detail by Kieffer *et al.* (2020 ▸). The obtained resolution of 0.013° is one order of magnitude better than that of typical laboratory diffractometers, and much faster (typically 5 to 10 min for an excellent signal-to-noise ratio). To further improve the resolution, we consider the acquisition of a 2D detector with smaller pixel size than that provided by the Pilatus 100k.

Interface specific X-ray diffraction techniques are a relatively new addition to the methods suitable for the characterization of interfacial processes. Through crystal truncation rod (CTR) diffraction and resonant anomalous X-ray reflectivity (RAXR) it is possible to selectively determine 3D interfacial structures with sub-Å resolution and elemental specificity. CTR diffraction is an extension of single-crystal X-ray diffraction focusing on a single crystal’s surface. Here, the translational symmetry breaks down, which means the Bragg conditions do not need to be met in the surface normal direction. Consequently, constructive interference is not limited to zero-dimensional Bragg peaks, but instead one-dimensional rods of intensity can be detected connecting these peaks in reciprocal space, albeit several orders of magnitude weaker than bulk Bragg peaks (Robinson & Tweet, 1992 ▸; Fenter, 2002 ▸). The measured intensity is extremely sensitive to changes in the structure of the interface region, which reaches from the bulk crystal to the undisturbed solution and includes any relaxed surface layers of the crystal, ordered absorbed water, and of course ions or particles registered at the interface. Due to the phase problem (Als-Nielsen & McMorrow, 2011 ▸), it is typically not possible to derive a structural model (electron density as a function of distance from the interface) from measured data. Instead, structural models are provided, and the corresponding CTR intensity is calculated. An interfacial structure can then be obtained by fitting the structural model to reproduce the measured data (Fenter, 2002 ▸).

More recently, CTR has frequently been combined with RAXR, which allows to determine the contribution of one or several resonant atoms to the total electron density as determined by CTR (Park *et al.*, 2005 ▸). The method is based on the dependence of the scattered or reflected intensity on the X-ray energy, close to the absorption edge of an element, if it is part of the interfacial structure. For RAXR the incident X-ray energy *E* is scanned around the absorption edge *E*_0_ of the resonant element at constant momentum transfer *q*. If the element is coherently registered at the interface, the energy scan will show modulations at *E*_0_ as superpositions of the element’s anomalous dispersion terms *f* ′ and *f* ′′ (see also Fig. 18), whose amplitude and phase will depend on the elements adsorbed quantity and average distance from the interface, respectively (Park & Fenter, 2007 ▸). Based on the CTR data, a model for the distribution of the resonant atom can then be constructed using similar approaches as for the CTR modeling. There are a number of related techniques based on the same physical effects, such as coherent Bragg rod analysis (COBRA) or diffraction anomalous fine structure (DAFS), all of which are in principle feasible at ROBL, but have not been implemented yet.

When hard X-rays (>7 keV) are applied, the method is suited for *in situ* investigations, *e.g.* of mineral interfaces in contact with a solution (Eng *et al.*, 2000 ▸; Fenter, 2002 ▸; Trainor *et al.*, 2004 ▸, 2006 ▸). The type of information obtainable through CTR/RAXR is valuable in the context of nuclear waste management, in order to characterize the interaction of radionuclides with mineral phases. As heavy scatterers with a high number of electrons, actinides are ideally suited elements, yet the publications remain relatively few, due to the lack of accessible beam time for this type of research, amongst other factors (Catalano *et al.*, 2005 ▸; Schmidt *et al.*, 2013 ▸). ROBL’s diffraction end-station is the only end-station purpose built for studying interfacial reactions of radioactive samples using surface X-ray diffraction techniques, such as CTR diffraction and RAXR. Several thin-film cell sample holders are available at the beamline, including triple confinement holders for radioactive (or very sensitive) samples based on more complex earlier designs (Schmidt *et al.*, 2011 ▸).

**Figure 16:**
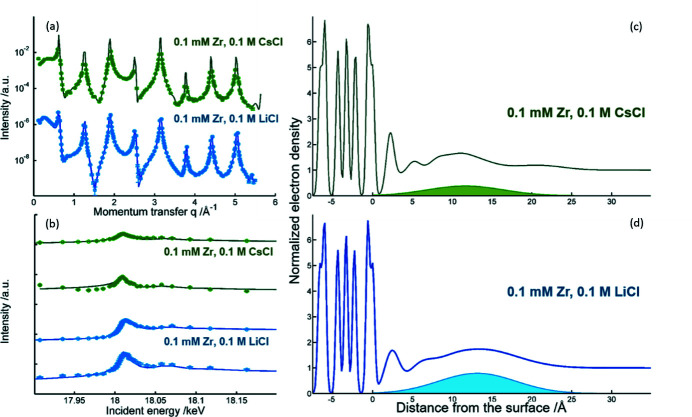
Growth of Zr nanoparticles on muscovite (001) from LiCl (blue) and CsCl (green) media. (*a*) Experimental CTR data (dots) and best fit models (lines). (*b*) Representative RAXR data at two values of *q* (dots) and best fit models (lines). (*c*, *d*) CTR-derived total electron density distributions (drawn lines) and RAXR-derived interfacial Zr electron densities (filled areas) above the mica (001) basal plane for the two media (Qiu *et al.*, 2018 ▸).

As an example, Fig. 16 ▸ shows representative data and the corresponding electron densities at the mica/water interface, after reacting muscovite with Zr for 24 h in two different background electrolytes (LiCl and CsCl) (Qiu *et al.*, 2018 ▸). The CTR-derived electron density changes abruptly at the mica surface (0 Å, defined as the position of the top-most oxygen layer in the mica structure) from the crystalline structure left to the aqueous phase right. The peak at about 2.5 Å corresponds to the first adsorbed water layer. The RAXR-derived electron density of Zr (filled area) is broadly distributed over 20–25 Å from the surface, indicating the presence of Zr-oxo-nanoparticles. From the CsCl medium, less Zr adsorbs (which is directly evident from the smaller amplitude in the RAXR modulations (Fig. 16 ▸, bottom left) and the formed nanoparticles are smaller. The result can be interpreted as an effect of the stronger competition by the more strongly adsorbing Cs^+^ relative to Li^+^, which in turn is caused by the weaker hydration of the large Cs^+^cation.

## 7. XRD-2 (Pilatus diffractometer)   

**Figure 17:**
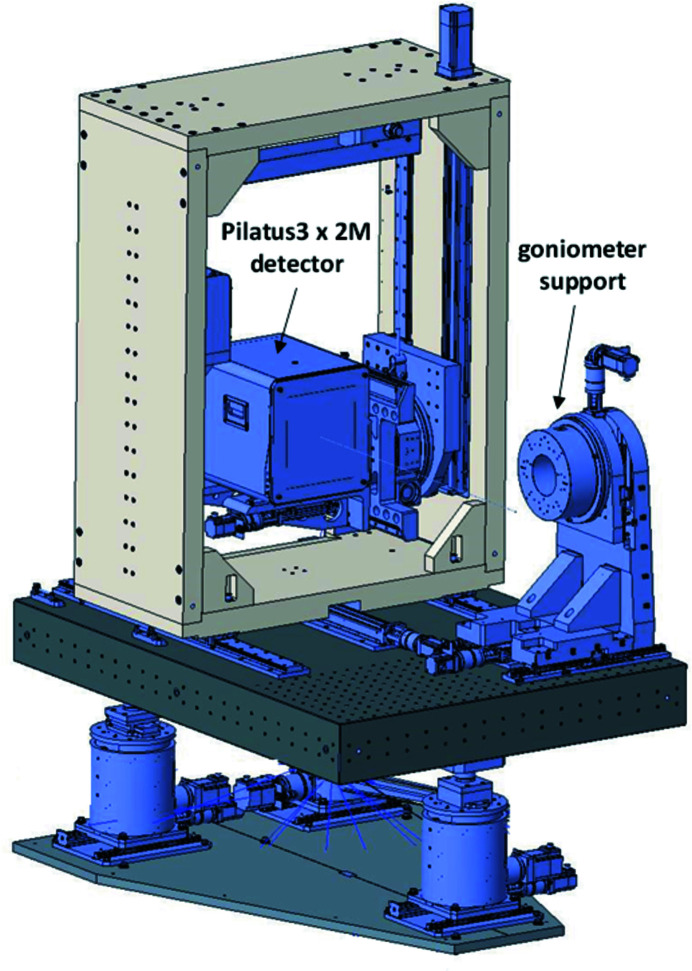
Pilatus diffractometer station (XRD-2).

Recent detector developments resulted in very large single photon-counting 2D detectors, which can be used for a complete registration of all reflexes in the reciprocal space in a short time, necessary for single-crystal diffraction of small- and large-molecule crystallography, as well as for the quick registration of complete scattering patterns during *in situ* measurements. While the large sensitive surface area of these detectors allows a simultaneous registration of a wide 2θ range, their heavy weight interferes with an accurate and reproducible movement relative to the sample using a six-circle diffractometer. For this reason, we decided to build a second diffractometer station, with a Pilatus 2M detector placed in a sturdy support frame which is mounted together with the sample goniometer on a common granite table. The design was developed by Instrument Design Technology Ltd together with SNBL/ESRF (Dyadkin *et al.*, 2016 ▸). The diffractometer is equipped with a Pilatus3 X 2M detector (Dectris Ltd), with a photon-sensitive layer of 450 µm-thick Si of an area of 253.7 mm × 288.8 mm (width × height), and a pixel size of 172 µm × 172 µm. The detector is mounted in a support frame, which allows lateral and vertical movements as well as rotation (see Fig. 17 ▸).

A total of 15 motorized movements allow the sample and the detector to be precisely positioned and aligned relative to the synchrotron beam. The granite table is a five-degrees-of-freedom system and consists of three 2.5 Mg-carrying high-stiffness jacks. Differential positioning of the custom guided jacks provides actuation of the vertical height (*z*), pitch and roll of the support frame. The jack assemblies are mounted on motorized horizontal slide units, that can be driven together to achieve a horizontal translation (*y*), or in opposition to each other to obtain a yaw movement. The detector mount comprises two parallel translational (*x*) and one vertical linear stage (*z*). The main linear translation supports the detector support frame. The second translation moves the detector support. The detector support is mounted on a goniometer 420 (Huber diffraction GmbH) which is used to rotate around the *y*-axis. The detector support unit, consisting of the Pilatus detector and two beam diagnostic devices, is counter-balanced by a 60 kg weight to support the vertical movement of the unit. The two beam diagnostic devices are positioned in the same translational plane as the photosensitive layer of the Pilatus detector and aid in shaping the X-ray beam. The first is an air beam viewer with a magnification of 0.8 (ESRF) and the second a FDS photonic direct beam camera (Photonic Science). The sample–detector distances can be varied between 140 and 600 mm. Three goniometers are available to mount samples: a Huber uniaxial goniometer 410, a Huber Kappa goniometer 512.410, and an Arinax Kappa goniometer MK3. Diffraction measurements can be combined with XANES and XRF spectroscopy using a single-element Si drift detector (Vortex X90 CUBE, 1000 µm SDD, 50 mm^2^ collimated down to 30 mm^2^, 25 µm Be window) with a FalconX1 processor. Parasitic scattering can be suppressed with additional collimators (titanium, tantalum or molybdenum) with an entry hole diameter of 2 mm and a total detection angle of 22.6°.

**Figure 18:**
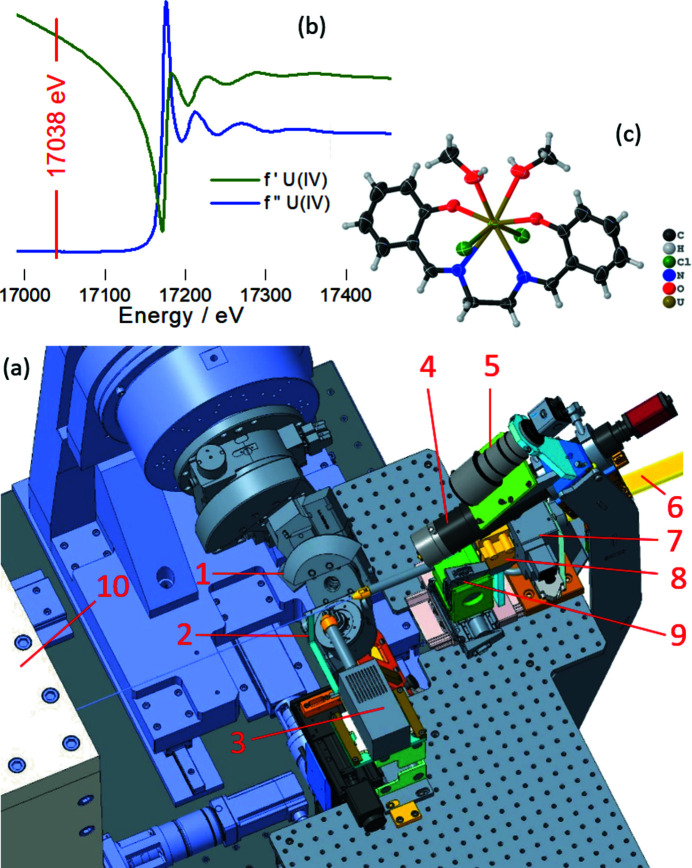
(*a*) Beam conditioning unit of XRD-2, the Pilatus diffractometer, with the Huber kappa goniometer mounted (1 – Kappa goniometer, 2 – motorized beam stop, 3 – Vortex SDD, 4 – long-distance microscope, 5 – macro camera, 6 – beam tube, 7 – entrance slits, 8 – ionization chamber (50 mm) to determine incoming beam intensity, 9 – motorized pin hole unit, 10 – support for Pilatus3 X 2M detector). (*b*) The fluorescence detector can be used to determine *f* ′ and *f* ′′ in the case of resonant measurements to select the appropriate excitation energy as shown here for uranium(IV). (*c*) This technique was applied to determine the structure of UCl_2_salen(MeOH)_2_, space group *P*4_3_2_1_2.

A beam conditioning unit in front of the goniometer (Fig. 18 ▸) comprises an entrance slit, a small ionization chamber to monitor the incoming beam intensity, and a collimation unit to suppress parasitic scattering from the slit blades. For optical sample alignment, a long-distance microscope K2 (Infinity Photo-Optical GmbH) is placed at a distance of 180 mm from the sample position. A macro camera is used to align supporting devices like a heat chamber HTK1200 (Anton Paar, up to 1200°C). The setup also includes a cryocooler 800 series (Oxford Cryosystems Ltd) which controls a temperature range of 80–400 K. The diffractometer is operated with the GUI-based software *Pylatus* (Dyadkin *et al.*, 2016 ▸).

Fig. 18 ▸ (top) shows the result of a feasibility test with a crystal of UCl_2_salen(MeOH)_2_ mounted in a sealed Kapton capillary. The excitation energy was set to 17038 eV to reduce the sample absorption and to avoid fluorescence arising from the uranium *L*_3_ absorption edge at 17162 eV. The fine structure of the anomalous scattering factors *f* ′ and *f* ′′ of U(IV) were extracted using the *kkcalc* code (Watts, 2014 ▸) from the U *L*_3_ absorption edge. The single-crystal diffraction patterns were registered with the program suite *Pylatus* (Dyadkin *et al.*, 2016 ▸) and the reflections were extracted using the *Crysalis* software (Rigaku). The structure was solved with *Olex2* (Dolomanov *et al.*, 2009 ▸) with a R1 of 2.99% (Radoske *et al.*, 2021 ▸).

**Figure 19:**
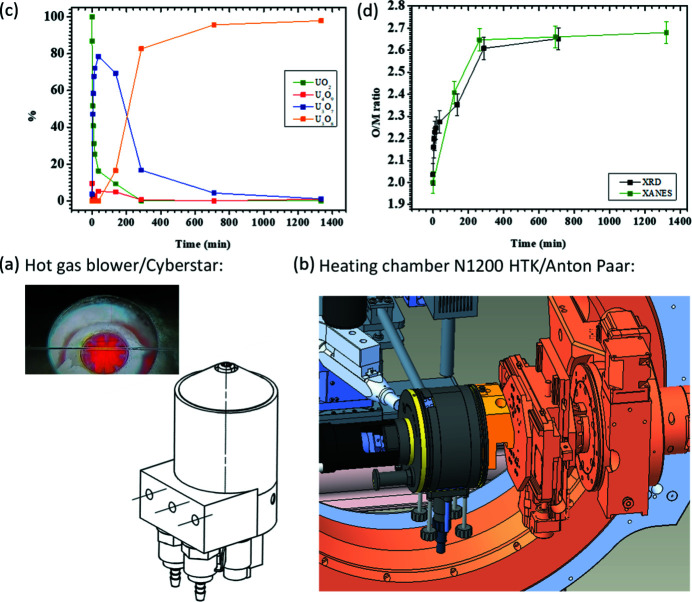
(*a*) Hot gas blower and (*b*) heating chamber N120HTK (Anton Paar). (*c*) Temporal evolution of the molar fractions of UO_2_, U_4_O_9_, U_3_O_7_ and U_3_O_8_ during the oxidation of UO_2_. (*d*) Temporal evolution of the O/M ratio during the oxidation of UO_2_.

*In situ* powder XRD is a powerful technique to follow structural modifications as a function of, for example, temperature or gas atmosphere, with various applications in kinetic, catalytic and thermodynamic studies. A hot gas generator from Cyberstar [Fig. 19 ▸(*a*)] is available to heat a sample up to 1000°C in air with a precision of ±1°C controlled by a Eurotherm and a gas flow controller. Alternatively, a heating chamber HTK1200N (Anton Paar) can be mounted at XRD-1 and XRD-2 [Fig. 19 ▸(*b*)]. This furnace can be used for measuring PXRD in reflection and transmission geometries at temperatures up to 1200°C in air, inert atmosphere (O_2_, N_2_, Ar, He, and other non-haza­rdous, non corrosive gasses) or in vacuum (≥10^−4^ mbar**)**. A capillary spinner enables measurements in transmission geometry for highly air-sensitive materials or samples with strong preferrential orientation.

Fig. 19 ▸(*c*) shows the stepwise transformation of UO_2_ into U_3_O_8_ through oxidation measured with the Pilatus diffractometer. Determining the molar fractions of the individual oxide phases (*i.e.* UO_2_, U_4_O_9_, U_3_O_7_ and U_3_O_8_) is essential for the understanding of oxidation reactions and their kinetics. Simultaneous with the PXRD measurements, the oxygen-to-metal ratio was determined by recording *in operando* the XANES spectra with the Vortex X90 CUBE silicon drift detector. Fig. 19 ▸(*d*) shows the good agreement between the O/M ratio derived from the PXRD oxide phase fractions and from the XANES spectra (De Bona *et al.*, 2021 ▸).

## 8. ROBL-II in comparison with other European radionuclide beamlines   

To the best of our knowledge, only five dedicated radionuclide beamlines are currently fully operational, all of them in Europe (Table 3 ▸). Although ROBL-II shares many characteristics with the other four beamlines, including a variety of techniques, its four permanently mounted experimental stations provide the possibility to use all available techniques even during a typical six-day run, with easy sample transport between the stations, which are in one controlled area. Other distinguished features are the high-count-rate detector and multisample cryostat for EXAFS, the six-crystal spectrometer with complete crystal sets for 1 and 0.5 m Rowland radii, and finally the surface and single-crystal diffraction stations for radioactive samples offered by XRD-1 and XRD-2.

The two KARA beamlines are distinguished by their proximity to dedicated alpha labs of INE, where sample preparation can be performed, as well as the possibility for *in situ* experiments at non-ambient conditions including heating up to 2000 K. Although beam time is no longer provided through the typical proposal review process, it is provided through collaboration with INE.

While these previously described beamlines have been designed with predominately alpha-emitting radionuclides in mind, the experimental stations of the MARS beamline incorporate substantial lead shieldings around samples to enable the measurement of much stronger gamma-emitting samples. Such lead-shieldings can also be (temporarily) established at the microXAS beamlines, which is further discriminated by its high spatial resolution through strong microfocussing down to 1 µm.

## Figures and Tables

**Table 1:** Typical radionuclides and their radioprotection-relevant parameters, including their maximum amount not to exceed the 185 MBq limit for both solids and liquids under RAD-2 condition (sample in underpressurized glovebox during measurements)

Nuclide (decay)	Half-life (years)	Specific activity (Bq mg^−1^)	Exemption limit (Bq)	Maximum amount (mg)
Tc-99 (β^−^)	2.1 × 10^5^	6.4 × 10^5^	1.0 × 10^7^	2.9 × 10^4^
Po-209 (α)	1.0 × 10^2^	6.2 × 10^8^	1.0 × 10^4^	3.0 × 10^−1^
Ra-226 (α)	1.6 × 10^3^	3.7 × 10^7^	1.0 × 10^4^	5.0 × 10^0^
Th-nat (α)	1.4 × 10^10^	8.2 × 10^0^	1.0 × 10^4^	2.3 × 10^7^
Pa-231 (α)	3.3 × 10^4^	1.7 × 10^6^	1.0 × 10^3^	1.1 × 10^2^
U-nat (α)	4.5 × 10^9^	2.6 × 10^1^	1.0 × 10^4^	7.1 × 10^6^
Np-237 (α)	2.1 × 10^6^	2.6 × 10^4^	1.0 × 10^3^	7.1 × 10^3^
Pu-239 (α)	2.4 × 10^4^	2.3 × 10^6^	1.0 × 10^4^	8.0 × 10^1^
Pu-242 (α)	3.7 × 10^5^	1.5 × 10^5^	1.0 × 10^4^	1.2 × 10^3^
Am-243 (α)	7.4 × 10^3^	7.4 × 10^6^	1.0 × 10^3^	2.5 × 10^1^
Cm-246 (α)	4.8 × 10^3^	1.1 × 10^7^	1.0 × 10^3^	1.7 × 10^1^
Cm-248 (α)	3.4 × 10^5^	1.6 × 10^5^	1.0 × 10^3^	1.2 × 10^3^
Bk-247 (α)	1.4 × 10^3^	3.8 × 10^7^	1.0 × 10^4^	4.9 × 10^0^
Cf-251 (α)	9.0 × 10^2^	5.8 × 10^7^	1.0 × 10^3^	3.2 × 10^0^

**Table 2:** Spherically bent striped crystal analyzers available

Analyzer	Bending radius (m)	Number of analyzers
Ge 111	0.5 and 1.0	5
Ge 220	0.5 and 1.0	5
Ge 100	0.5 and 1.0	5
Ge 620	0.5 and 1.0	5
Si 111	0.5 and 1.0	5
Si 220	0.5 and 1.0	5
Si 331	0.5	5
Si 311	0.5	1
Si 951	0.5	1

**Table 3:** Comparison of ROBL-II with other dedicated radionuclide beamlines

	ROBL-II	INE-KARA	ACT-KARA	Mars-SOLEIL	microXAS-SLS
Reference	This paper	(Rothe *et al.*, 2012 ▸)	(Zimina *et al.*, 2016 ▸, 2017 ▸)	(Llorens *et al.*, 2014 ▸; Sitaud *et al.*, 2012 ▸)	(Borca *et al.*, 2009 ▸)
Source	SBM (0.6 T) on 6 GeV ring at 200 mA	BM (1.6 T) on 2.5 GeV ring at 200 mA	Superconducting 2.5 T wiggler on 2.5 GeV ring at 200 mA	BM (1.7 T) on 2.75 GeV ring at 500 mA	Minigap in-vacuum undulator
Photon flux	6 × 10^12^ ph/s at 20 keV and 200 mA	2 × 10^10^ ph/s at 18 keV	10^11^ ph/s at 20 keV and 100 mA	1 × 10^12^ ph/s at 12 keV and 430 mA	3 × 10^12^ ph/s at 12 keV
Mirror 1	VCM† (Si, Rh, Pt) at 2.5 mrad	VCM (Rh) at 2.7 mrad)	VCM (Si, Rh, Pt), mirror-less	VCM (Si, Pt)	Horizontally deflecting toroid (Rh)‡
DCM	Si(111), Si(111)_30°_, Si(311) multilayer	Si(111), Si(311), Ge(422), InSb(111)	Si(111), Si(311)	Si(111) and Si(220) on sagittal bender for horizontal focusing	Si(111), Si(311), Ge(111)
Mirror 2	Double-toroid (Rh, Pt), VFM§ (Si)	Toroid (Rh)	Double-toroid (Si, Rh), VFM (Ir), mirror-less	VFM (Si, Pt)	Elliptical Kirkpatrick–Baez (KB) (Rh)
Energy range (keV)	3.5–35	2.1–25	3.4–55	3.5–35	4–23
Spot size on sample (h × v) (µm)	21 × 69 (focused) 10000 × 2000 (unfocused)	500 × 500	1000 × 1000 (focused)	300 × 300 15 × 15 (with additional KB optics)	1.0 × 1.0 with 10^11^ ph/s dynamic focusing
Maximum activity	185 MBq	Exemption limit ×10^6^ for non-fissile isotopes, 200 mg for fissile ^235^U and ^239^Pu		185 GBq, 18.5 GBq per sample	100 × LL per sample (Swiss clearance limit) maximum 10 samples
Maximum dose rate	0.5 µSv h^−1^ outside of glovebox, 15 µSv h^−1^ in contact with sample confinement	None, as long as the annual dose rate of 6 mSv for controlled areas is respected		<0.5 µSv h^−1^ outside the beamline, <2 mSv h^−1^ inside the beamline	0.5 µSv h^−1^ outside of hutch, 2 mSv h^−1^ in experimental hutch
Experimental stations	(A) EXAFS with 18-element Ge (Mirion) and Falcon-X (XIA) (B) Spectrometer (Johann-type, five-crystal, 0.5 and 1 m Rowland circle) (C) Six-circle diffractometer (LaB6 (110: 0.013°FWHM) (D) Pilatus diffractometer	EXAFS with Vortex ME4 and Vortex EX60 (Hitachi), xMAP-DXP (XIA)	Spectrometer (Johann-type, 5-bent-crystals, 1 m Rowland circle) with Si(Li) SDD (Ketek) and Vortex EX60 or EXAFS with 8-element HPGe array detector (Mirion), xMAP-DXP (XIA)	(A) High-resolution XRD (B) EXAFS with 13-element Ge (ORTEC) and DXP-xMAP or 13-element SDD (MIRION) with Xspress 3 (Quantum) or Spectrometer (Johann-type, 4-crystal 0.5 m, and 1-crystal 1 m), Image plate (MAR345) or Hybrid pixel CdTe (Pilatus /DECTRIS) for 2DXRD and SAXS	(A) Micro-XAS with 1-element (Ketek) and 5-element (SGX) SDD, Falcon-X (XIA), Eiger 4M area detector or Spectrometer (von Hamos)
In operation since	1998 (ROBL), 2020 (ROBL-II)	2005	2017	2009–2010 (below exemption limit), 2013	2006–2007
User access	Public user facility (33%), inhouse and collaborations (67%)	Inhouse and collaborations		Public user facility (47%), CEA dedicated time (33%), inhouse (20%)	Public user facility

† VCM: vertical collimating mirror.

‡ Vertical collimation and dynamic focusing in horizontal.

§ VFM: vertical focusing mirror.
